# BrassicaTED - a public database for utilization of miniature transposable elements in *Brassica* species

**DOI:** 10.1186/1756-0500-7-379

**Published:** 2014-06-20

**Authors:** Jayakodi Murukarthick, Perumal Sampath, Sang Choon Lee, Beom-Soon Choi, Natesan Senthil, Shengyi Liu, Tae-Jin Yang

**Affiliations:** 1 Department of Plant Science, Plant Genomics and Breeding Institute, and Research Institute for Agriculture and Life Sciences, College of Agriculture and Life Sciences, Seoul National University, Seoul 151-921, Republic of Korea; 2 PHYZEN Genome Institute, 501-1, Gwanak Century Tower, 1808 Nambusunhwan-ro , Gwanak-gu, Seoul 151-836, Korea; 3 Genomics and Proteomics Laboratory, Centre for Plant Molecular Biology & Biotechnology, Tamil Nadu Agricultural University, Coimbatore, Tamilnadu, India; 4 The Key Laboratory of Oil Crops Biology and Genetic Breeding, The Ministry of Agriculture, Oil Crops Research Institute, the Chinese Academy of Agricultural Sciences, Wuhan 430062, China

**Keywords:** *Brassica*, Miniature inverted-repeat transposable element (MITE), Terminal-repeat retrotransposon in miniature (TRIM), Miniature form transposable elements (mTEs), Short interspersed elements (SINEs), TE Database

## Abstract

**Background:**

MITE, TRIM and SINEs are miniature form transposable elements (mTEs) that are ubiquitous and dispersed throughout entire plant genomes. Tens of thousands of members cause insertion polymorphism at both the inter- and intra- species level. Therefore, mTEs are valuable targets and resources for development of markers that can be utilized for breeding, genetic diversity and genome evolution studies. Taking advantage of the completely sequenced genomes of *Brassica rapa* and *B. oleracea*, characterization of mTEs and building a curated database are prerequisite to extending their utilization for genomics and applied fields in *Brassica* crops.

**Findings:**

We have developed BrassicaTED as a unique web portal containing detailed characterization information for mTEs of *Brassica* species. At present, BrassicaTED has datasets for 41 mTE families, including 5894 and 6026 members from 20 MITE families, 1393 and 1639 members from 5 TRIM families, 1270 and 2364 members from 16 SINE families in *B. rapa* and *B. oleracea,* respectively. BrassicaTED offers different sections to browse structural and positional characteristics for every mTE family. In addition, we have added data on 289 MITE insertion polymorphisms from a survey of seven *Brassica* relatives. Genes with internal mTE insertions are shown with detailed gene annotation and microarray-based comparative gene expression data in comparison with their paralogs in the triplicated *B. rapa* genome. This database also includes a novel tool, K BLAST (Karyotype BLAST), for clear visualization of the locations for each member in the *B. rapa* and *B. oleracea* pseudo-genome sequences.

**Conclusions:**

BrassicaTED is a newly developed database of information regarding the characteristics and potential utility of mTEs including MITE, TRIM and SINEs in *B. rapa* and *B. oleracea.* The database will promote the development of desirable mTE-based markers, which can be utilized for genomics and breeding in *Brassica* species. BrassicaTED will be a valuable repository for scientists and breeders, promoting efficient research on *Brassica* species. BrassicaTED can be accessed at http://im-crop.snu.ac.kr/BrassicaTED/index.php.

## Findings

### Background

Transposable elements (TEs) are ubiquitous and compose a large fraction of most of eukaryotic genomes. For example, more than 80% of the maize genome [1] and 40% of the *B. rapa* genome are occupied by transposons [2]. TEs can be mobilized and accumulated in the genome through cut and paste mechanism or a replicative transposition mechanism. Transposition of TEs can cause gene knock-out or rearrangement, altered regulation of expression, acquisition of novel function, and/or gene structure modification [3-5]. Although plant genomes have large numbers of TEs, most of them remain as non-autonomous TEs, which cannot transpose without the assistance of a “partner” element providing transposition enzymes, because of accumulation of mutations, incomplete transposition processes or genetic and epigenetic control [4,6]. Miniature form transposable elements (mTEs) are especially small (<800 bp) TEs that are present in high copy numbers. They lack functional coding genes and rely on *trans*-activation by long and complete counterpart autonomous TEs [3,7-9]. The mTEs include the terminal repeat retrotransposon in miniature (TRIM), short interspersed element (SINE) and miniature inverted-repeat transposable element (MITE) [4,8-10] families.

TRIMs are derivatives of retrotransposons from which the coding domain for transposition has been lost. TRIMs have terminal direct repeats (TDRs) (100–250 bp) flanked by 5-bp target site duplications (TSDs) and contain an internal sequence that begins with the signature region of the primer binding site (PBS) of tRNA-methionine and ends with a polypurine tract (PPT) motif [8,11,12]. Studies have suggested that amplification and mobilization of TRIM elements are controlled by autonomous partner elements like LTR retrotransposons. TRIMs have been identified in various plant species and are present at moderately high levels, with 2000 copies, in the *Brassica* genome [8,11,13]. TRIM element-based markers have been effective for high quality mapping in *Brassica*[11].

Short interspersed elements (SINEs) are non-autonomous, non-LTR retrotransposons that are transcribed by RNA polymerase III and widely distributed in eukaryotic organisms [10,14]. SINEs have unique structural features, with so-called head, body and tail sequences, and have been found in higher copy numbers in animals than in plants [15-17]. For instance, the Alu SINE family is present in > 1,500,000 copies in the human genome and covers > 11% of the total genome [14,18,19]. Propagation, maintenance and movement of SINEs are controlled by long-interspersed nucleotide elements (LINEs). To date, 210 SINE families from various plant and animal genomes including 16 SINE families related to Brassicaceae have been reported in SINEBase [20].

MITEs are small, non-autonomous class II DNA transposons that are AT-rich and have DNA structures characteristic of TEs, like terminal inverted repeats (TIRs) and TSD of variable lengths. Based on their TSD, most MITEs have been classified into several superfamilies. *Stowaway* superfamily members recognize 2-bp (TA) nucleotides and duplicate these at their terminal region via insertion. Similarly, *Tourist* superfamily members produce unique TAA duplications, *hAT* superfamily members produce 5-, 6-, or 8-bp TSD, *Mutator* superfamily members produce 9- or 10-bp TSD, and *En/Spm* superfamily members produce 3-bp non-unique TSD [4]. Available evidence suggests that MITEs originate through either internal deletion or cross mobilization from autonomous transposable elements [3]. MITEs can amplify into high copy numbers, although the MITEs are thought be mobilized through cut-and-paste mechanisms. Analysis of complete information for MITEs on a genome-wide scale will be important for a better understanding of how MITE transposition can produce high copy numbers, as well as of their influence on genome evolution [21].

mTEs are stably maintained and highly dispersed throughout the genome, often in association with genic regions, where they can alter gene structure and function [22,23]. Insertion of MITEs has been reported to cause changes in pigmentation and flowering time in potato and rice, respectively [24,25]. However, most mTE insertions may not cause phenotypic changes but can alter gene expression and epigenetic patterns [8,26-28]. Their high copy number and close association with gene-rich regions make mTEs a potential source of molecular markers, mTE-based marker systems have been successfully developed in various species for genetic mapping as well as analysis of genetic diversity, genome-wide association and evolution [8,29-34].

The genus *Brassica* (family Brassicaceae) is composed of more than 3700 species including important vegetable and oil-seed crops such as kimchi-cabbage, cabbage, and oilseed rape (*B. rapa, B. oleracea* and *B. napus*) [35]. *B. rapa* was the first *Brassica* vegetable to be sequenced and annotated, revealing recent genome triplication at 13–17 MYA, after divergence from *Arabidopsis thaliana* 20 MYA [2]. While only 6% of the *A. thaliana* genome is occupied by TEs [36,37], around 38-40% of the *B. rapa* and *B. oleracea* genome is derived from TEs [2]. Genome analysis has suggested that TEs are important players not only in terms of genome size increases but also for gene and genome evolution, especially in the recently triplicated *Brassica* genome [2]. Recently, a plant MITE database was created for 41 sequenced plant genomes, and 174 MITE families from *B. rapa* were identified using *in silico* MITE identification tools [38]. However, experimental validation of identified mTE families and genome-scale characterization of their members with biological utility for *Brassica* species have not been developed or included in any databases. Furthermore, there is no resource with information regarding the distribution of TRIM, SINE and MITE families in various genomic regions for the *Brassica* species. Therefore, we aimed to establish a unique web portal containing detailed characterization of TEs in *Brassica* species. As a starting point, we have investigated the MITE, TRIM and SINE families of *B. rapa* and *B. oleracea* at the whole-genome scale and developed a database, BrassicaTED (Brassica Transposable Elements Database), for effective utilization of MITE, TRIM and SINEs in the *Brassica* genome.

### Construction and content

**Figure 1:**
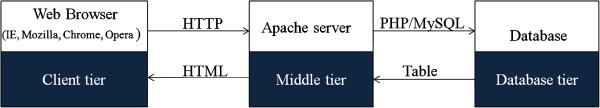
**Three-tier architecture of BrassicaTED.** The client tier receives input from users and sends the request to an Apache server. The middle tier receives the request, processes it and passes it to the database tier through PHP and MySQL. The user request is extracted from MySQL as a table and displayed on a browser in the html page.

We developed BrassicaTED as a database to provide comprehensive information about mTEs in the *B. rapa* and *B. oleracea* genomes. This database has three-tier architecture, namely a client tier, a middle tier and a database tier (Figure 1). The user-friendly interface, i.e. the client tier, was developed using PHP (v4.3.9) and JavaScript [39-41]. In the database tier, all information related to the mTEs is stored in a MySQL (v4.1.20) database. In the middle tier, an application forces all HTTP requests to be processed by an Apache web server (Figure 1). The database is currently hosted on a CentOS (5.8) Linux operation system. Additionally, we included the NCBI BLAST algorithm (version: 2.2.15) for sequence-based searches and sequence extractor tools.

### Browse

**Figure 2:**
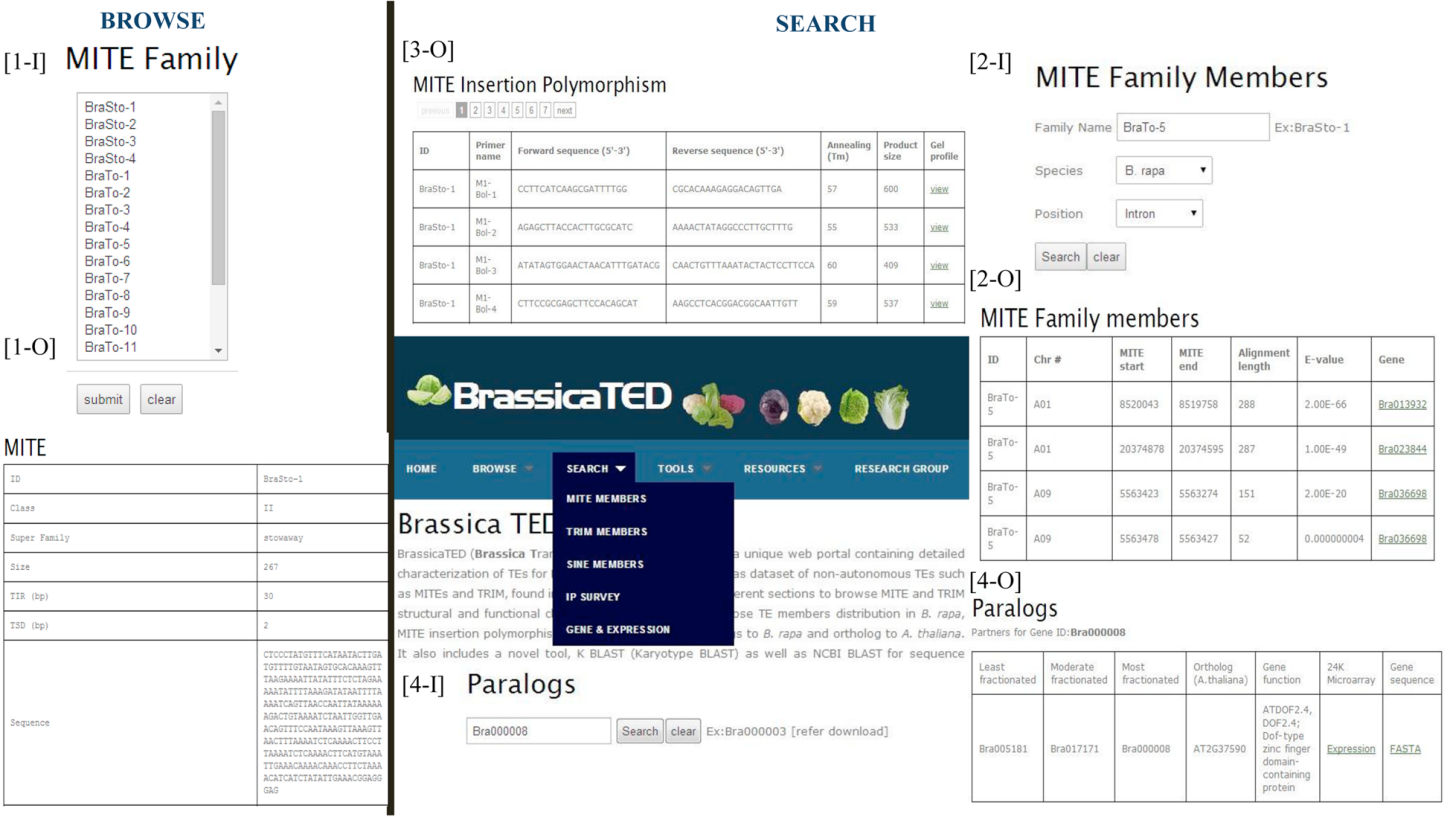
**Display of the Browse and Search panels of BrassicaTED. [1-I]** The Browse page for the MITE family listing characterized MITEs. **[1-O]** Output display of the selected MITE family. **[2-I]** Query page to retrieve MITE family members on each chromosome. **[2-O]** Result display of MITE member page. **[3-O]** Display of all MITE families and MITE insertion polymorphism. **[4-I]** Query box for Paralogs & Expression tab. **[4-O]** Paralog partners and ortholog gene display for selected genes.

In the Browse section, BrassicaTED provides a convenient panel in which to browse the structural characteristics, family classification, and sequence information with copy numbers in *B. rapa* and *B. oleracea* and *A. thaliana* for mTEs. It currently contains information for 20 MITE families, 5 TRIM families and 16 SINE families characterized in the whole genomes of *B. rapa* and *B. oleracea*. We developed this section to be fully updatable as more mTEs are identified and characterized (Figure 2 [1-I]).

### Search

The Search section was developed to allow exploration of annotation and analysis for each mTE family in detail. In this section, there are five sub-categories: MITE Members, TRIM Members, SINE Members, IP Survey, and Gene & Expression (Figure 2).

### MITE members

*B. rapa* pseudo-chromosome sequences with unanchored scaffolds (283 Mb) and gene annotation information (version 1.2) were obtained from the publically available database [42]. We included 20 MITE families that were identified from our studies and others in *Brassica*[8,30,43-45]. Consensus representative sequences of the 20 MITE families were used to identify their members from 283 Mb *B. rapa* pseudo-chromosome sequences. The physical position of each MITE on the *B. rapa* pseudo-chromosome was described using a custom Perl script. A total of 5894 MITEs (Br-members) were recently identified in the *B. rapa* genome [45]. Among them, 98% could be classified based on the type of genomic sequence in which they were located, such as intergenic region, 5’- or 3’-UTR, exon or coding sequence (CDS), and intron, whereas the remaining 2% were located on unannotated scaffolds of the *B. rapa* genome.

The recent availability of 385 Mb of *B. oleracea* pseudo-chromosome sequences (version 1.0) [46,47] allowed that analysis to expand to the *B. oleracea* genome. Similar to *B. rapa,* a total of 6026 members (Bo-Members) were annotated based on physical position and gene annotation information for *B. oleracea*[42]. This work revealed that compared with Br-members, very few Bo-members (three) are present in the CDS of *B. oleracea*[45].

### TRIM members

In a previous study, we characterized four TRIM families (TRIM of Brassicaceae -1, -2, -3, -4) and their members in 96 Mb of *B. rapa* BAC end sequences and 434 Mb of *B. oleracea* shotgun sequences, [8]. Now, we additionally characterized a high copy family, TB-5 (Cassandra), in the pseudo-chromosome sequences of *B. rapa* and *B. oleracea*[13]. TRIM element insertion was analyzed based on physical position information and the annotation of the genomes of *B. rapa* and *B. oleracea*. In total, 1393 and 1639 copies from the five TRIM families were identified from *B. rapa* and *B. oleracea* whole-genome pseudo-chromosome sequences, respectively (Table 1)*.* Distribution analysis of these five TRIM families shows insertion throughout the chromosomes of the *B. rapa* and *B. oleracea* genomes. Interestingly, 619 (44%) and 656 (40%) members reside in or within 2 kb of a gene in the *B. rapa* and *B. oleracea* genome, respectively. This information will be highly valuable for marker development and characterization of target regions that have TRIM element insertion.

**Table 1:** **Characteristics of five TRIM families in the *B****.* ***rapa *** **and *B****.* ***oleracea *** **genomes**

**TRIM family**	**No. of TRIM members in *B. rapa *** **(283 Mb) genome**								**No. of TRIM members in *B. oleracea *** **(385 Mb) genome**							***Copies in A. thaliana *** **(119 Mb)**
	**Total**	**Intergenic space**^**a**^	**Near gene**^**b**^	**Elements in genic regions**				**Un mapped**^**d**^	**Total**	**Intergenic space**^**a**^	**Near gene**^**b**^	**Elements in genic regions**				
				**5’ UTR**^**c**^	**CDS**	**Intron**	**3’ UTR**^**c**^					**5’ UTR**^**c**^	**CDS**	**Intron**	**3’ UTR**^**c**^	
TB-1	206	71	54	12	1	39	17	12	273	164	61	17	0	19	12	65
TB-2	319	147	92	15	2	35	18	10	321	162	110	11	0	29	9	16
TB-3	52	22	7	4	3	8	4	4	51	25	15	3	1	3	4	0
TB-4	351	157	78	14	7	37	14	44	392	235	106	19	2	16	14	0
TB-5	465	233	96	8	1	40	13	74	602	397	150	21	0	13	21	96
Total	1393	630	327	53	14	159	66	144	1639	983	442	71	3	80	60	177

^a^MITE members categorized by their insertion >2 kb from the nearest gene.

^b^MITE members categorized by their insertion 500-2000 bp from the nearest gene.

^c^MITE members residing in the 500 bp upstream (5’UTR) or downstream (3’UTR) of the start or stop codon, respectively.

^d^MITE members found in unanchored scaffolds of the *B. rapa* genome.

### SINE members

We have included the characterization of the members of 16 SINE families from Brassicaceae, which we have named as SINE of Brassicaceae 1–16. The sequence information for 16 families was obtained from SINEBase, and 1270 and 2364 members were retrieved from the *B. rapa* and *B. oleracea* genomes [20]. The insertion of SINEs in various genomic locations was characterized based on physical position and genome annotation information, showing that 599 (47.1%) and 1154 (48.8%) of the members from *B. rapa* and *B. oleracea*, respectively, were present in close association with genic regions (with < 2 kb of a gene) (Table 2). This will be a valuable resource from which researchers can develop markers for tightly linked genes or regions on the chromosome.

**Table 2:** **Characteristics of 16 SINE families in the *B****.* ***rapa *** **and *B****.* ***oleracea *** **genomes**

**SINE family**	**No. of SINE members in *B. rapa *** **(283 Mb) genome**								**No. of SINE members in *B. oleracea *** **(385 Mb) genome**							**Copies in *A. thaliana *** **(119 Mb)**
	**Total**	**Intergenic space**^**a**^	**Near gene**^**b**^	**Elements in genic regions**				**Un mapped**^**d**^	**Total**	**Intergenic space**^**a**^	**Near gene**^**b**^	**Elements in genic regions**				
				**5’ UTR**^**c**^	**CDS**	**Intron**	**3’ UTR**^**c**^					**5’ UTR**^**c**^	**CDS**	**Intron**	**3’ UTR**^**c**^	
SB-1	3	3	0	0	0	0	0	0	98	49	32	7	0	4	6	0
SB-2	43	2	0	2	0	38	1	0	59	8	4	0	0	45	2	132
SB-3	134	70	36	6	0	12	6	4	278	156	80	20	0	9	13	4
SB-4	0	0	0	0	0	0	0	0	0	0	0	0	0	0	0	38
SB-5	155	72	54	6	0	11	7	5	375	205	99	23	0	18	30	3
SB-6	248	134	71	12	0	7	14	10	394	213	132	21	0	7	21	28
SB-7	372	202	106	17	0	11	20	16	355	197	119	16	0	2	21	3
SB-8	0	0	0	0	0	0	0	0	80	28	26	14	0	2	10	0
SB-9	141	62	49	7	0	6	12	5	201	102	69	11	0	7	12	0
SB-10	16	5	10	0	0	0	0	1	134	67	48	7	0	6	6	0
SB-11	4	4	0	0	0	0	0	0	56	22	25	3	0	2	4	0
SB-12	10	5	3	0	0	2	0	0	101	46	37	7	0	5	6	0
SB-13	1	0	1	0	0	0	0	0	95	43	30	7	0	7	8	0
SB-14	142	69	43	9	0	12	8	1	137	74	37	10	0	6	10	0
SB-15	1	1	0	0	0	0	0	0	1	0	0	0	1	0	0	0
SB-16	0	0	0	0	0	0	0	0	0	0	0	0	0	0	0	4
Total	1270	629	373	59	0	99	68	42	2364	1210	738	146	1	120	149	212

^a^MITE members categorized by their insertion >2 kb from the nearest gene.

^b^MITE members categorized by their insertion 500–2000 bp from the nearest gene.

^c^MITE members resid in the 500 bp upstream (5’UTR) or downstream (3’UTR) of the start or stop codon, respectively.

^d^MITE members found in unanchored scaffolds of the *B. rapa* genome.

### Insertion polymorphism (IP) survey

**Figure 3:**
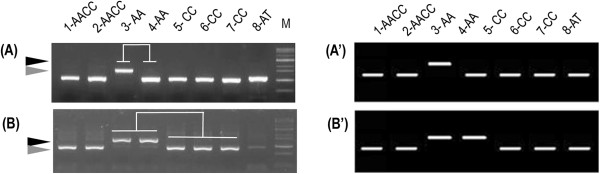
**MITE insertion polymorphism analysis shows intra- and inter- species polymorphism between *B. rapa *and *B. oleracea.*** Representative analysis of intra **(A)**- and inter **(B)**- species polymorphism between *B. rapa* and *B. oleracea*. The original gels are shown in **A** and **B**, whereas **(A’)** and **(B’)** show the corresponding images generated based on the gel scores. The accessions used for the MIP survey are the same as those in [30].

Insertion polymorphism inter- and intra- *Brassica* species due to insertion or absence of a mTE in specific accessions was surveyed by PCR using flanking primers [48]. For MITE insertion polymorphism (MIP) analysis, 187 and 145 MITE targets were surveyed from *B. rapa* and *B. oleracea* members, respectively [30,45]. Among them, primers for 162 Br-members and 127 Bo-members produced clear amplicons, of which over than 52% (150) showed MIP in the tested *Brassica* relatives and their MIP information were summarized such as Figure 3. BrassicaTED includes information for the primers used and polymorphism profiles with the tested accessions. Data for additional insertion polymorphism surveys of more MITE members and for TRIM and SINE members will be added in the future.

### Gene and expression analysis

BrassicaTED includes information regarding paralogous genes in *B. rapa* and their orthologs from *A. thaliana*, obtained from the *B. rapa* genome database [42]. Single, duplicate, and triplicate paralogs and their orthologs in *A. thaliana* are listed according to the gene order in *A. thaliana.* The expression level of *B. rapa* genes with internal MITE insertion was surveyed against the *B. rapa* 24 k microarray database, which was generated from four different stress treatments: cold (4°C), salt (250 mM NaCl), drought (air-dry) and ABA (100 μM) [49].

### Tools

**Figure 4:**
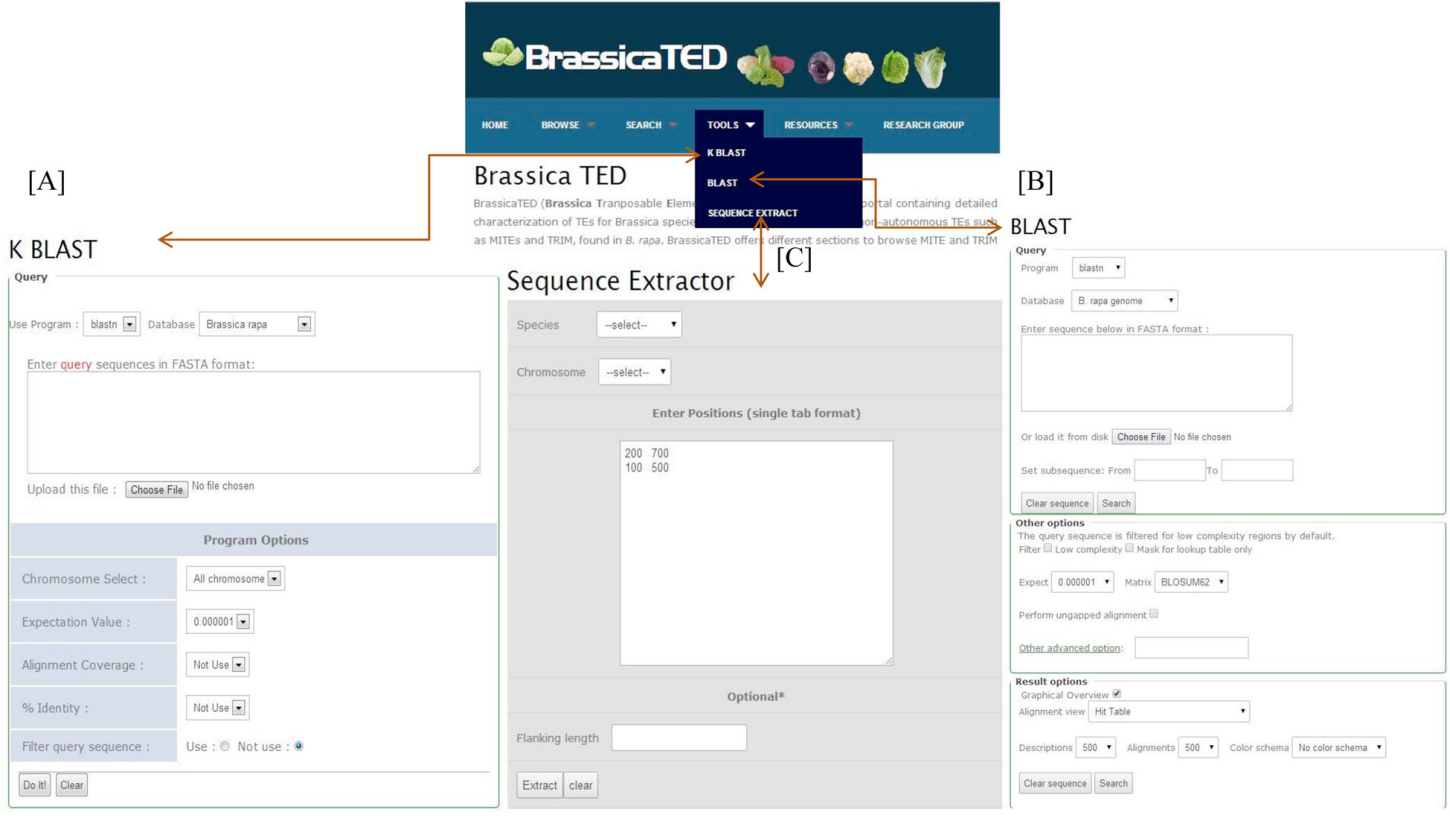
**Display of tools panel.** Display of K BLAST [**A**] and NCBI BLAST [**B**] and Sequence Extractor [**C**].

This section includes tools for basic bioinformatics analysis to visualize or map the query sequence onto the chromosome region and perform similarity searches. A sequence extractor tool is also provided to extract target sequences for further analysis, such as for MIP primer development or related gene structure identification (Figure 4).

### K BLAST

We developed a new tool, which we call K BLAST (Karyotype BLAST), to map the unique query sequence (s) onto homologous chromosomal regions and visualize the resulting distribution pattern. Users can visualize the locations of many different query sequences on each chromosome in different colors using K BLAST. It is a straightforward approach to inspect the distribution or dispersion of any DNA sequences (e.g. genes or repeats) on the chromosomes of *B. rapa* and *B. oleracea*. We have also included *A. thaliana* genome sequence for use in K BLAST.

### BLAST

The tool panel offers the standard NCBI BLAST program for sequence similarity searches against the genome sequences. Users can match nucleotide or protein sequence (s) against database sequences such as whole-genome sequences of *B. rapa*, *B. oleracea*, and *A. thaliana*.

### Sequence extract

We incorporated a sequence extractor tool using Python script. This can be utilized by entering single or multiple start and end positions in column tab-delimited format in the input query box for specific regions on the selected chromosome of *B. rapa* and *B. oleracea*. This tool also provides an optional input box to add flanking region length for input start and end positions.

### Utility and discussion

TEs are important factors affecting genome size and genome evolution. Among those, miniature form TEs (mTEs) are valuable targets for genomics and phylogenetic studies. Comprehensive analysis and tools are desirable for effective utilization of mTEs for molecular studies, and were lacking. We developed BrassicaTED web as an interface for extensive characterization of MITEs, TRIM and SINEs in the *Brassica* genome. We will keep adding newly identified mTE elements to our database.BrassicaTED provides three sections (Browse, Search, and Tools) with user-friendly navigation. First, there are three parts of the Browse section: one each for MITEs, TRIMs and SINEs. Users can choose any MITE family in the list box and click ‘submit’ to enter the area pertaining to that family (Figure 2 [1-I]). This tab retrieves a summary of all MITE families included, such as MITE element classification, sequence information, structure, total members of a particular family with navigation to view the members and their closely related members, etc. (Figure 2 [1-O]). Similarly, TRIM and SINE family members can also be queried to obtain the family characterization by choosing any element from that family section. The Browse panel thus allows users to find summaries of complete information about families of MITEs, TRIMs and SINEs.In the Search panel, users can type the name of any family in the search box to find its members. Using the position drop-down option, users can filter the members returned by choosing the required genomic location (intergenic, CDS, intron, UTR) and clicking ‘Search’ for retrieval (Figure 2 [2-I]). This retrieves members of the selected family in each chromosome, their start and end positions on the chromosome, the length of the query alignment and e-value threshold, obtained from annotation (Figure 2 [2-O]). When ‘intergenic’ is chosen, the names of the two genes flanking the element are returned. When other options like CDS, intron, 5′UTR and 3′UTR are chosen, the member located in the corresponding genic regions is given with the gene name.

*Brassica* genomes contain triplicated homologous counterparts relative to the genome of *A. thaliana*[2,50]*.* Recent investigation of BraSto-2 suggested that MITEs may have important roles in triplicated *Brassica* genome evolution [30]. BrassicaTED also provides navigation tools to determine paralogous partners and microarray-based gene expression profiles of individual mTE-inserted genes (Figure 2 [2-I]). Transposition of MITEs, TRIM and SINEs in various genomic locations can alter the gene structure and also gene expression patterns [8,30,44]. mTEs associated with genes may cause gene silencing, sub-functionalization or neo-functionalization. The information regarding mTE position and annotation of genes harboring mTE insertion will be helpful for association studies and candidate gene approaches by comparison of their functional diversity among a range of accessions.

The Search panel also includes IP survey, which is a valuable source for genomics, genetic diversity studies, and evolutionary analysis [31,51,52]. Currently, there are about 18349 IP targets from 41 mTEs known in the *Brassica* genome and >50-85% of them are related to genic regions. The accumulated IP marker information from 289 MITE targets (Figure 2 [3-O]) will be effectively used for *Brassica* genomics studies. We surveyed 289 MIP targets that provided good genetic diversity based on presence or absence of a MITE in the target region among different accessions. MIP survey profiles in seven different *Brassica* derivatives were scored as 1, 2 and 3 for insertion as a full site, non-insertion as an empty site and both insertion and non-insertion as corresponding full and empty sites, respectively. The gel analysis has also been included as a link and users can view the gel profile with graphical images and accession information by clicking the link (Figure 3). This information will help researchers choose MIP primers for diversity studies using their own population. We will continue to update our database with more IP analysis of mTE targets. We also welcome other researchers to submit their data regarding mTEs of *Brassica* species.

The next sub-section of the Search panel is ‘Gene & Expression’. As a mesopolyploid species, the *B. rapa* genome has been estimated to contain 41,174 protein coding genes in triplicated form compared to the genome of *A. thaliana*[2]. Our study on BraMi-1 MITE insertion suggests that MITEs may modify the structure of one copy among such triplicated genes and eventually its expression pattern [8,30]. The ‘Gene & Expression’ analysis provides information regarding paralogous genes and 24 k microarray expression profiles for any gene of *B. rapa*. Under this sub-section, users can find differential expression patterns of mTE-inserted genes via comparison with expression of their paralogs. Users can obtain the available paralogous partner and gene sequences in FASTA format for any *B. rapa* gene by entering the gene ID (Figure 2 [4-I]). Clicking the ‘Expression’ link navigates to another page where the user can select expression profiles derived from four stress treatments: cold, salt, drought and ABA (Figure 2 [4-O]). The expression level of the selected genes will be shown with output as a graph or a table. This feature will be updated with more microarray datasets and can be used to analyze expression differences for any gene of interest.

**Figure 5:**
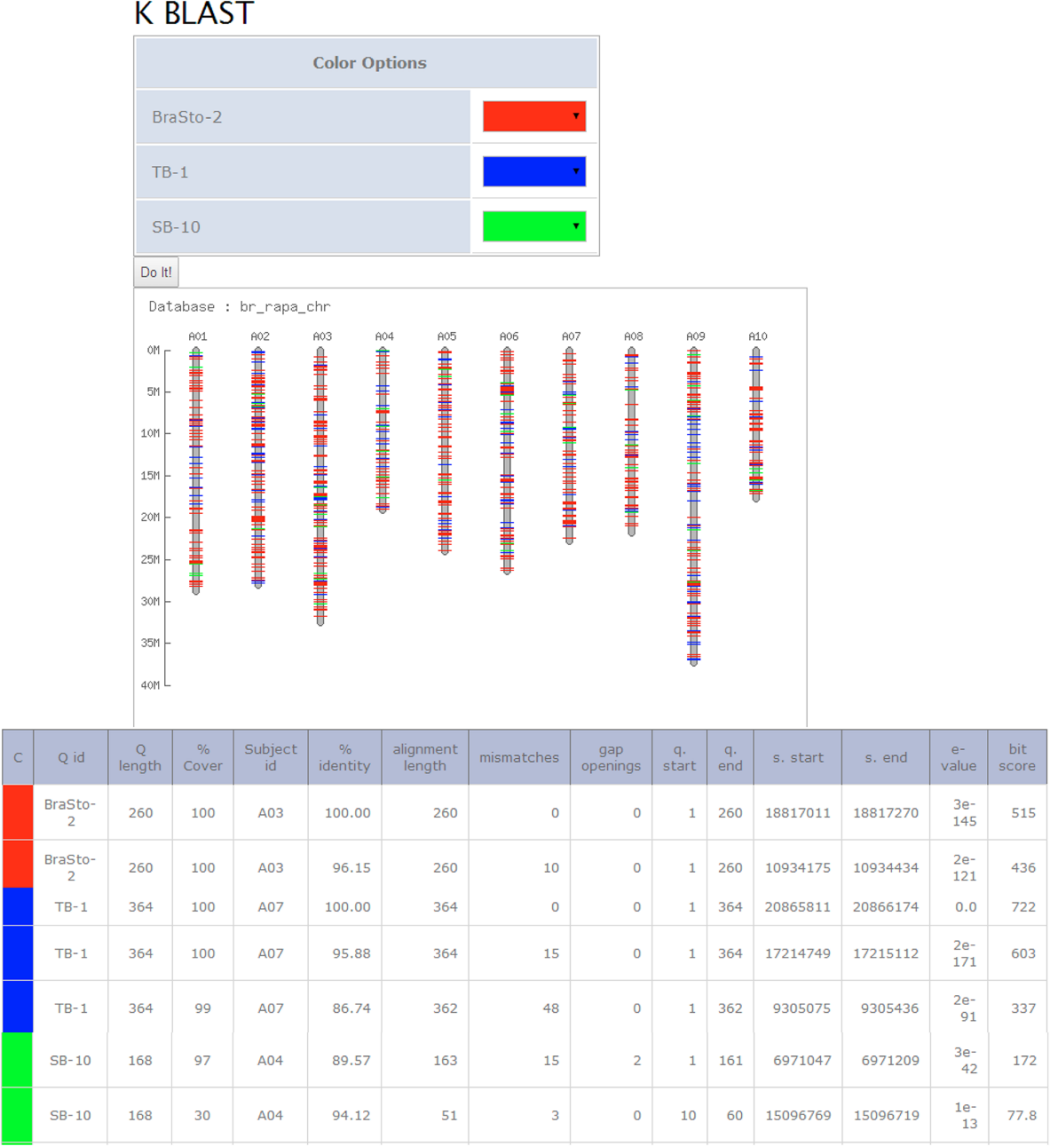
**Output display of the K BLAST tool using three mTE families**.

In the Tools section using K BLAST, users can visualize the distribution of query sequences with user-defined specific options. This program contains options similar to NCBI BLAST but it has been updated with several new features (Figure 4 [A]). Users can perform K BLAST searches against all of the chromosomes or any particular chromosome of *B. rapa*, *B. oleracea*, or *A. thaliana* by selecting the chromosome number in the ‘Chromosome Select’ option. Furthermore, users can filter the output based on alignment coverage and sequence identity from each query by choosing a range between 60 and 100%. In K BLAST, up to 25 queries can be visualized on chromosome sequences simultaneously. An example shows the K BLAST output using three different mTE families (BraSto-3 TB-1, and SB-10) (Figure 5). The NCBI BLAST algorithm retrieves the matched database sequences in a flat file format, and even though it displays a graphical representation, it cannot show the overall positions precisely, unlike K BLAST. In addition, K BLAST offers options for filtering the query coverage and the percentage of sequence identity, which can be highly useful for research.BrassicaTED also offers a sequence extractor tool. With this tool, the user can select the chromosome number in the drop-down menu, input the start and end position(s) in the input text box, and click ‘Extract’ to retrieve the sequences. This returns an output of extracted sequences in FASTA format with defined positions. We have provided an example of the input position on the program as reference for the users (Figure 4 [C]). This tool will be useful for users wanting to extract target region(s) with flanking sequence for designing primers or further analyses.

In sum, we have characterized members of 41 mTE families using the whole-genome pseudo-chromosome sequences of *B. rapa* and *B. oleracea*, which allowed us to develop a database to promote effective utilization of TEs. Previously developed databases for *B. rapa* such as BRAD mostly handle distribution and mining of whole-genome data [42]. Similarly, the *B. oleracea* genome database (Bolbase) [46] was designed to provide genomic data, which mainly includes whole genome sequence, annotation and synteny comparison with *B. rapa* and *A. thaliana*. Though recently reported database for plant MITEs, P-MITE, has data for MITE families from 41 plant species, it does not provide specific information regarding MITE insertion positions in the *B. rapa* and *B. oleracea* genomes. By contrast, BrassicaTED is built to establish an interactive web platform for all types of mTEs and includes in-depth analysis of 41 mTE families in *B. rapa* and *B. oleracea*, as well as in *A. thaliana*. In addition, BrassicaTED provides tools to extract gene sequences and compare the expression of paralogous genes using the 24 k microarray datasets.

## Conclusion

BrassicaTED is a new database exclusively made with mTEs of *Brassica* species, especially from *B. rapa* and *B. oleracea*. This will be an important repository to promote the utilization of mTEs and the elucidation of the effects of mTEs on genome evolution in *Brassica* species. Unlike other publically available *Brassica* databases, BrassicaTED has a unique user-friendly visualization tool (K-BLAST) and a microarray expression data comparison tool for *B. rapa*. BrassicaTED will be a valuable storehouse for scientists and breeders who work on *Brassica* species and will be continuously updated as more data is uncovered.

## Availability and requirements

Datasets in BrassicaTED are freely accessible for research purposes for non-profit and academic organizations at http://im-crop.snu.ac.kr/BrassicaTED/index.php. The database is optimized for Internet Explorer, Mozilla Firefox, Google Chrome and Safari.

## Competing interests

The authors declare that they have no competing interests.

## Author’s contributions

PS and TJY developed the methodology and conducted the study. JM, PS, SCL and BSC participated in the database and bioinformatics tool development. JM, PS, NS, SL and TJY drafted the manuscript, which was revised by all authors. All authors read and approved the final manuscript.
